# Workplace loneliness and job turnover: a 6-month prospective study

**DOI:** 10.1093/joccuh/uiaf009

**Published:** 2025-02-03

**Authors:** Natsu Sasaki, Kanami Tsuno, Reiko Kuroda, Kotaro Imamura, Hisashi Eguchi, Akihito Shimazu, Norito Kawakami

**Affiliations:** Department of Mental Health, Graduate School of Medicine, The University of Tokyo, 7-3-1 Hongo, Bunkyo-ku, Tokyo, 113-0033, Japan; School of Health Innovation, Kanagawa University of Human Services, Research Gate Building TONOMACHI2, 3-25-10, Tonomachi, Kawasaki-ku, Kawasaki-shi, Kanagawa, 210-0821, Japan; Division for Environment, Health, and Safety, The University of Tokyo, 7-3-1 Hongo, Bunkyo-ku, Tokyo, 113-0033, Japan; Department of Digital Mental Health, Graduate School of Medicine, The University of Tokyo, 7-3-1 Hongo, Bunkyo-ku, Tokyo, 113-8655, Japan; Department of Mental Health, Institute of Industrial Ecological Sciences, University of Occupational and Environmental Health, Seigaoka 1-1, Yahatanishi-ku, Kitakyushu, Fukuoka, 807-8555, Japan; Faculty of Policy Management, Keio University, 5322 Endo, Fujisawa, Kanagawa, 252-0882, Japan; Department of Digital Mental Health, Graduate School of Medicine, The University of Tokyo, 7-3-1 Hongo, Bunkyo-ku, Tokyo, 113-8655, Japan

**Keywords:** turnover, occupational health, organizational commitment, psychosocial factors at work, retention, social isolation

## Abstract

**Objectives:**

This longitudinal study examined the associations of workplace loneliness with job turnover at 6-month follow-up among Japanese full-time employees.

**Methods:**

This study employed a 6-month prospective design as part of the Employee Cohort Study in Japan. Data from wave 15 (February 2024; baseline, T1) and wave 16 (August 2024; follow-up, T2) were used. Only participants who were employed at baseline and completed the follow-up survey were included in the analysis. Workplace loneliness at T1 was measured using 3 scales: the Loneliness at Work Scale (LAWS), a single-item workplace loneliness scale, and a 3-item scale developed by modifying the short UCLA (University College, Los Angeles) Loneliness Scale for workplace loneliness. Job turnover at T2 was defined based on responses indicating either leaving a job or moving to a different company within the preceding 6 months. Multivariable logistic regression analysis assessed the association between workplace loneliness and job turnover, adjusting for sociodemographic variables.

**Results:**

In total, 706 employees who were employed at baseline were included in the analysis. Observed turnover at follow-up was *n* = 47 (6.7%). Participants who experienced turnover had significantly higher baseline scores for LAWS and the 3-item scale (*P* = .044 and *P* = .012, respectively). In the multivariable logistic regression, all 3 workplace loneliness scales demonstrated similar and significant associations with turnover at follow-up.

**Conclusions:**

Workplace loneliness leads to job turnover. Further research is needed to address the generalization and explore mechanisms of the present findings.

## Teaser

Key points

- Workplace loneliness, a work-related dimension of loneliness, has been reported to have detrimental effects on employee health and well-being and organizational productivity.
- Although workplace loneliness should be associated with job turnover, which could result in a huge loss of productivity for the organization, no previous study has examined the prospective association between workplace loneliness and job turnover.

- This study found that workplace loneliness, as measured by 3 scales of workplace loneliness, was significantly and positively associated with job turnover at 6-month follow-up among Japanese full-time employees.

- This study suggests that workplace loneliness is a risk factor for future job turnover. It provides a rationale for companies/organizations to consider preventing workplace loneliness in order to reduce productivity losses due to employee turnover.

## Introduction

Workplace loneliness is a work domain–specific dimension of loneliness and is defined as a “distress caused by the perceived lack of good quality interpersonal relationships in a work environment.”^1^ The British Red Cross Survey reported that workplace loneliness is common: approximately 1 out of 10 workers often experience workplace loneliness in the United Kingdom.^2^ Similar to general loneliness, workplace loneliness is associated with mental health and well-being; it is further associated with work-related outcomes, such as deteriorated job satisfaction, work engagement, organizational commitment, and work performance of workers.^3^^-^^6^

Turnover of employees is an important work-related outcome for employers because employee turnover requires cost and effort to compensate for the increased workload of the co-workers in the same workgroup, recruit a new employee, and so on.^7^^-^^9^ Research by the Co-op and New Economics Foundation found that loneliness costs UK employers £2.5 billion a year.^10^ These costs are comprised of the impacts of loneliness on worker turnover (64%, £1.62 billion), well-being and productivity (26%), caring responsibilities (9%), and ill health and associated absence (1%). Turnover is expected to explain a large portion of the impact of loneliness on employer costs. However, this expectation is based on general loneliness, not workplace loneliness. Moreover, the expectation stems from research of well-being and turnover, not of loneliness and turnover. Determining the impact of workplace loneliness on employer costs through increased turnover requires studying the association between workplace loneliness and turnover. A limited number of previous empirical studies have reported that workplace loneliness is associated with turnover intention.^11^^,^^12^ Qualitative studies have reported that workplace loneliness may lead to actual turnover.^13^^,^^14^ General loneliness, not only workplace loneliness, has been associated with unemployment^15^ as well as turnover intention.^16^ However, to date, no quantitative study has examined the association between workplace loneliness and future turnover.

Previous studies have used a variety of approaches to measure workplace loneliness. One popular approach is to use a scale designed specifically to measure loneliness in the workplace, such as the Loneliness at Work Scale (LAWS),^1^^,^^17^ which asks about emotional deprivation at work and lack of social companionship at work. The other approach is to use a well-established general loneliness scale, such as the University of California, Los Angeles Loneliness Scale (V.3), Short Form 3-item (UCLA-LS3-SF3),^18^ to measure workplace loneliness, which assesses loneliness indirectly by asking about interpersonal circumstances at work.^2^ The third approach is to directly ask about feelings of loneliness at work.^2^ These approaches may capture different aspects of loneliness at work, and thus may lead to different findings. It would be useful to use multiple measures to know if there was a robust association between workplace loneliness and job turnover across the measures.

The present longitudinal study examined the associations of workplace loneliness with job turnover at 6-month follow-up among Japanese full-time employees, by using a well-established scale^1^ as well as other scales to measure workplace loneliness to ascertain the robustness of the findings.

## Methods

### Study design and subjects

This 6-month prospective study was conducted as a part of the Employee Cohort Study conducted during the COVID-19 pandemic in Japan (E-COCO-J). Full-time employees were recruited from the registered panel of the Japanese online survey company. This online cohort was established in March 2020 (*n* = 1448) using the online survey company, and the survey was administered repeatedly to those participants.

This particular study retrieved the data from wave 15 of the survey in February 2024 as a baseline (T1) and from wave 16 of the survey in August 2024 as a follow-up (T2). This was because wave 15 was the first survey including scales of workplace loneliness in the cohort study. Participants working at baseline who responded to the follow-up survey were included in the analysis. The Research Ethics Committee of the Graduate School of Medicine/Faculty of Medicine, The University of Tokyo [no. 10856-(2)(3)(4)(5)], approved this study.

### Measurements

Scales of workplace loneliness were measured in February 2024 (T1), and job turnover was measured in August 2024 (T2). Workplace loneliness was assessed using 3 different scales: LAWS,^1^^,^^17^ a single-item scale of the overall evaluation of workplace loneliness, and a 3-item scale developed based on a scale of general loneliness adopted for the workplace.

#### LAWS

Participants were asked to complete the LAWS on their perceptions of loneliness in the workplace over the preceding 4 weeks.^1^^,^^17^ The LAWS includes 16 items. Nine items relate to emotional deprivation (eg, “I often feel abandoned by my co-workers when I am under pressure at work”), and 7 items relate to social companionship (eg, “There is no one at work I can share personal thoughts with if I want to”). Participants rated all items on a 7-point Likert scale from 1 (strongly disagree) to 7 (strongly agree). Nine items were reversed items, so the scores were inverted accordingly. Thus, possible scores ranged from 16 to 112. Higher scores indicated greater loneliness at the workplace. The Japanese version of the scale was developed by the translation and back-translation process and was reported to have acceptable levels of internal consistency and reliability, and factor-based and structural (hypothesis-testing) validity.^19^ In this survey, the Cronbach a of the Japanese LAWS was .932 (*n* = 706).

**Table 1:** Characteristics of participants who worked at baseline (T1) and responded at follow-up (T2) (*n* = 706).

**Variables [possible range]**	***n* (%)**	**Mean (SD) [min, max]**
**Age, y**		47.6 (10.0) [26, 64]
**Younger than 30**	23 (3.3)	
**30-39**	142 (20.1)	
**40-49**	199 (28.2)	
**Over 50**	342 (48.4)	
**Sex**		
**Men**	393 (55.7)	
**Women**	313 (44.3)	
**Marital status**		
**Single**	310 (43.9)	
**Married**	396 (56.1)	
**Education** ^**a**^		
**High school**	172 (24.4)	
**Some college**	157 (22.2)	
**University**	308 (43.6)	
**Graduate school**	36 (5.1)	
**Unknown**	33 (4.7)	
**Occupation**		
**Manager**	87 (12.3)	
**Nonmanual**	410 (58.1)	
**Manual/other/unknown**	209 (29.6)	
**Company size** ^**b**^		
**1000+ employees**	221 (31.3)	
**300-999**	98 (13.9)	
**50-299**	190 (26.9)	
**<50**	162 (22.9)	
**Unknown**	35 (5.0)	
**Industry** ^**c**^		
**Manufacturing**	160 (22.7)	
**Medical and welfare**	89 (12.6)	
**Retail and wholesale business**	70 (9.9)	
**Public sector**	65 (9.2)	
**IT services**	55 (7.8)	
**Finance, insurance, real estate**	55 (7.8)	
**Transportation/construction**	54 (7.7)	
**Life-related services and entertainment**	50 (7.1)	
**Professional and technical services**	48 (6.8)	
**Education and learning support**	32 (4.5)	
**Restaurant, hotel business**	11 (1.6)	
**Primary industry**	2 (0.3)	
**Others**	15 (2.1)	
**Employment contract**		
**Permanent**	557 (78.9)	
**Temporary**	149 (21.1)	
**Loneliness at Work Scale (LAWS) [16-112]**		57.82 (17.81)
**Single-item scale of workplace loneliness [0-3]**		0.63 (0.89)
**3-item scale of workplace loneliness [3-9]**		4.64 (1.77)

a The data were collected in May 2020 or August 2020.

b The data were collected in May 2020.

c The data were collected in February 2022.

#### Single-item scale of workplace loneliness

Participants were asked how often they felt lonely in the workplace to rate the frequency of feeling workplace loneliness over the preceding month after reading the following definition of loneliness: “Loneliness is the subjective feeling of being alone. You may feel lonely even when you are connected to others. We would like to ask you about your situation over the past month.” The question was adopted from the British Red Cross Survey.^2^ The response options were modified to a 4-point Likert scale: 0 (almost never), 1 (sometimes), 2 (often), and 3 (almost always). Higher scores indicated greater workplace loneliness. This scale was not validated. The Pearson correlation coefficient for LAWS was 0.525 (*P* < .001).

#### Three-item scale of workplace loneliness

We developed a 3-item scale of workplace loneliness by modifying the UCLA-LS3-SF3,^18^ following the approach taken by the British Red Cross Survey.^2^ We used the Japanese translation of these 3 items^20^ and added the wording “in your workplace or at work” to the beginning of each of the following items: “How often do you feel that you lack companionship?” “How often do you feel left out?” and “How often do you feel isolated from others?” All items were rated on a 3-point Likert scale: 1 (hardly ever), 2 (some of the time), and 3 (often).^18^ Thus, possible scores ranged from 3 to 9. Higher scores indicated greater workplace loneliness. In this survey, the Cronbach a of the 3-item scale was .817 (*n* = 706). The Pearson correlation coefficient for LAWS was 0.697 (*P* < .001).

#### Job turnover

Job turnover was assessed by using 2 original items at 6-month follow-up. One of the items asked if a respondent had left their job and was currently unemployed at the follow-up point (unemployment). The other item identified whether a respondent had changed jobs and moved to another company/organization in the preceding 6 months (changed employer). Those who answered affirmatively to either response were defined as having experienced job turnover.

#### Demographics

The sociodemographic characteristics included sex, age, educational attainment, marital status, occupation, industry, company size, and employment contract.

### Statistical analysis

Student *t* test was used to assess differences in the workplace loneliness scores between those who had experienced turnover and those who had not. A multivariable logistic regression analysis was conducted to assess the relationship of workplace loneliness with job turnover after 6 months by adjusting for sex, age, marital status, occupation, and employment contract. The 3 scales of workplace loneliness were examined individually.

Statistical significance was set as a 2-sided *P* < .05. SPSS 28.0 Japanese version (IBM) was used.

## Results

In total, 907 participants (62.6% of the initial sample) responded at the baseline (T1) and 810 (response rate 55.9%) responded to the follow-up survey (T2). After excluding those with missing responses, the 706 employees employed at baseline were included in the analysis. Participants’ characteristics are presented in Table 1. About half of participants were over 50 years old. The majority were men (56%), married (56%), and nonmanual workers (58%) who had earned a university degree (44%).

A comparison of the mean scores of the 3 scales of workplace loneliness at baseline between participants who had experienced turnover at follow-up and those who had not is presented in Table 2. The LAWS and the 3-item scale of workplace loneliness scores at baseline (T1) were higher for those who had experienced turnover at follow-up (*P* = .044 and *P* = .012, respectively). No significant difference was found in the single-item scale of workplace loneliness.

**Table 2:** Comparison of mean scores of scales of workplace loneliness at baseline between respondents who left the company (turnover) and those who stayed at the same company at follow-up.

**Scales [possible score range]**	**Turnover at follow-up (*n* = 47)**	**Stayed at the same company (*n* = 659)**	***P* for difference (*t* test)** ^**a**^
	**Mean (SD)**	**Mean (SD)**	
**LAWS [16-112]**	62.89 (17.40)	57.46 (17.79)	.044*
**Single-item scale of workplace loneliness [0-3]**	0.89 (1.03)	0.62 (0.88)	.077
**3-item scale of workplace loneliness [3-9]**	5.36 (1.97)	4.59 (1.75)	.012*

^a^*P < .05. LAWS: Loneliness at Work Scale.

The multivariable logistic regression result is presented in Table 3. After adjusting for the covariates, all 3 scales showed significant positive associations with turnover at 6 months. The adjusted odds ratio (aOR) for an increase of one point in the score was 1.02 (95% CI, 1.00-1.03) for LAWS, 1.35 (95% CI, 1.01-1.82) for the single-item scale of workplace loneliness, and 1.24 (95% CI, 1.07-1.45) for the 3-item workplace loneliness scale. In a similar multivariate logistic regression of unemployment status at follow-up (a component of job turnover; *n* = 12), the LAWS, the single-item, and the 3-item scales were not significantly associated with not working at follow-up: aOR = 1.00 (95% CI, 0.97-1.04), 1.25 (95% CI, 0.65–2.42), and 1.22 (95% CI, 0.87–1.70), respectively. However, these scales were significantly associated with changing employer at follow-up (*n* = 36): aOR = 1.02 (95% CI, 1.00-1.04), 1.46 (95% CI, 1.06-2.02), and 1.27 (95% CI, 1.07-1.51), respectively. The 3 scales’ associations with turnover were significant even when the sample was limited to participants who were 58 years old or younger (*n* = 568), meaning that, at follow-up, they had not yet reached the mandatory retirement age in Japan (ie, 60 years old).

**Table 3:** Adjusted odds ratio for turnover at follow-up among Japanese employees using multivariable logistic regression analysis (*n* = 706).

	**aOR**	**95% CI**	**aOR**	**95% CI**	**aOR**	**95% CI**
**LAWS**	1.02	1.00-1.03	—		—	
**Single-item scale of workplace loneliness**	—		1.35	1.01-1.82	—	
**3-item scale of workplace loneliness**	—		—		1.24	1.07-1.45
**Sex (ref.; men)**	1.40	0.71-2.75	1.38	0.70-2.73	1.38	0.70-2.73
**Age, y**	1.02	0.98-1.05	1.02	0.99-2.73	1.02	0.98-1.05
**Marital status (ref.: married)**	1.19	0.63-2.26	1.15	0.60-2.21	1.12	0.58-2.14
**Occupation (ref.: manager)**						
**Nonmanual**	1.30	0.41-4.07	1.27	0.40-3.99	1.34	0.43-4.20
**Manual/other/unknown**	1.31	0.41-4.26	1.32	0.41-4.27	1.40	0.43-4.53
**Employment contract (ref.: permanent)**	1.38	0.70-2.74	1.43	0.72-2.83	1.41	0.71-2.79

Abbreviation: aOR: adjusted odds ratio. LAWS: Loneliness at Work Scale.

a For scales of workplace loneliness, aORs for an increase in score of 1 are shown.

## Discussion

This longitudinal study showed that workplace loneliness was significantly associated with job turnover at the 6-month follow-up. Three different workplace loneliness scales presented similar results, showing the robustness of the findings. Workplace loneliness was associated with changing employers, but not with becoming unemployed; this result should be carefully interpreted because of the small number of turnover cases.

Previous empirical studies have reported that workplace loneliness is associated with turnover intention,^11^^,^^12^ which is a strong determinant of actual turnover. Qualitative studies have reported that workplace loneliness may lead to actual turnover.^13^^,^^14^ The present finding is in line with these previous research findings. To our knowledge, the present study provides the first quantitative evidence of the association between workplace loneliness and future turnover.

When we calculated an aOR for a 1 SD increase in the workplace loneliness score, the score was quite similar among the scales of workplace loneliness: 1.42 for the LAWS; 1.31 for the single-item scale; and 1.46 for the 3-item scale. The 3 scales seem to have a similar predictive value for turnover at the 6-month follow-up, although the 3-item scale was slightly more predictive. These 3 scales were developed to capture different aspects of workplace loneliness. For instance, the LAWS measures emotional deprivation at work and lack of positive relationships at work whereas the 3-item scale asks about the current situation of workplace social isolation, and the single-item scale asks participants to evaluate their workplace loneliness. These 3 scales were only moderately correlated with each other. Nevertheless, they may measure a common core element of workplace loneliness that could affect turnover. It is suggested that there is a robust association between workplace loneliness and job turnover, regardless of different approaches to measure workplace loneliness.

The OR of turnover among those who reported “almost always” (score of 3) compared with those who reported “almost never” (score of 0) on the single-item scale of workplace loneliness may be estimated as 2.46 (=1.35^3). This result is lower than the relative risk of turnover associated with loneliness (4 times) used in the estimation of loneliness-related costs among UK employers.^10^ The financial impact of workplace loneliness may be half of their estimation. However, the present study was conducted in Japan, where companies maintain a longer-term employment policy and workers less frequently migrate from one company to another compared with the United States and European countries.^21^ The impact (OR) of workplace loneliness on turnover may be smaller than in other countries because of Japan’s labor market culture.

### Limitations

This study has several limitations. Although the distributions of sex and age of the study participants were similar to those of the working population of Japan, the proportion of participants with a university degree or higher in this study was 49%, which is higher than the national average of approximately 37% for workers in Japan.^22^ Thus, the generalizability of the findings may be limited. Also, as this cohort study was conducted in Japan, it may not be generalized to other countries. Given that the Japanese financial year ends in March and many people change jobs in April, it is essential to examine whether conducting the study during a different research period might yield different results. The single-item and the 3-item scales of workplace loneliness were not fully validated. The measurement of actual turnover was also made by original self-report questions. There may be random measurement errors that led to the underestimation of the association. Attrition (dropout) bias should be considered as 89% of baseline respondents participated in the follow-up. The present sample size did not permit conducting more sophisticated analyses, such as adjusting for psychosocial work environment (eg, workplace support) or testing the mediation effect of workplace loneliness on the association between psychosocial work environment and job turnover. Future large-scale studies are needed to address these issues. In addition, there may be unmeasured confounding factors, such as personality. This study did not test the mediation effect of turnover intention, although turnover intention was regarded as a precursor of actual turnover. However, a strength of this study is that it examined the impact of workplace loneliness with actual turnover as a work-related outcome, which may matter in a company’s business operation.

### Conclusion

Workplace loneliness was found to be a significant predictor of actual job turnover at the 6-month follow-up. Employers and managers need to be aware of workplace loneliness to reduce turnover among employees. Future research should be conducted to confirm the finding in other settings and to explore mechanisms such as mediators and moderators of the effect of workplace loneliness on turnover.

## Data Availability

The data supporting this study’s findings are available from the corresponding author, N.K., upon reasonable request.
